# Oral Lichen Planus and Mutated *TP53*—A Road to Cancer?

**DOI:** 10.3390/dj10090176

**Published:** 2022-09-16

**Authors:** William Peter Holbrook, Helga M. Ögmundsdottir

**Affiliations:** 1 Faculty of Odontology, University of Iceland, Vatnsmýrarveg 16, 101 Reykjavík, Iceland; 2 Faculty of Medicine, School of Health Sciences, University of Iceland, Vatnsmyrarvegur 16, 101 Reykjavík, Iceland

**Keywords:** oral lichen planus, TP52, oral squamous cell cancer

## Abstract

The malignant potential of oral lichen planus (OLP) has been discussed and disputed for decades. The lesions are often characterized by strong expression of the TP53 protein in the basal layer of the mucosa. In 2002, we reported the presence of *TP53* mutations in nine out of 27 OLP lesions tested. At follow-up in 2009, one case of oral squamous cell cancer (OSCC) had occurred in a different site six years later. In contrast, in another case, *TP53* mutation persisted for years without malignant transformation. In a longitudinal study of eight selected patients with OSCC or different pre-malignant lesions, it was concluded that *TP53* mutations could occur early or late in the development of OSCC. A follow-up in the present, almost 20 years later, revealed that one further case of OSCC had occurred in a *TP53*-mutated case of OLP, 21 years after the first sample was taken, again in a different site. With this second case, this small study now points towards a risk of developing OSCC in *TP53*-mutated OLP lesions. A review of recent literature indicates a growing consensus that OLP should be regarded as a potentially pre-malignant lesion. Several protein markers have been studied, but none proved useful for prediction of malignant progression. The great majority of published studies are retrospective, and it has been suggested that multi-centre prospective studies will be needed to reach a definitive answer on the malignant potential of OLP, and particularly, to identify contributing factors. Screening for TP53 mutations could help to identify the subgroup of OLP patients that is truly at risk of developing oral cancer.

## 1. Introduction

Oral lichen planus (OLP) has been associated with a risk for developing oral squamous cell carcinoma (OSCC) for decades. If this risk exists, it is not very high, and this is reflected in the long-lasting debate and discussion on the subject [1,2,3,4]. As with other lesions that are proposed to lead to cancer development, the most important question is which patients are going to progress to malignancy and whether they can be identified so that preventive measures might be applied. Interestingly, the top results of simple Google searches for “premalignant lesions” and “premalignant lesions markers” are oral lesions and oral squamous cell carcinoma. One obvious reason is, of course, that these lesions are easily observable. Lessons learnt from studying the path leading to OSCC will have implications for other sites in the body.

Cancer research was revolutionized by the application of molecular biology, leading to the discovery of thousands of genes associated with the development of cancer and to the identification of molecular markers that are clinically useful for indicating cancer risk or progression. *TP53* is famously known as the gene most frequently mutated in cancer, and its multitude of functions give ample reason for its relevance in cancer development [5]. When we published our first paper in 2002, the main emphasis was on its role in “protecting the genome” i.e., responding to DNA damage or stress linked directly to DNA damage, leading either to temporary cell cycle arrest to give time for DNA repair or to programmed cell death if repair failed [6]. In the meantime, TP53 has been shown to be central to the response to a great variety of cellular stresses, including metabolic and oxidative stress [5]. *TP53* mutations can occur early or late in the carcinogenic process, depending to some extent on the tissue of origin, with early mutations being observed in ecto- and mesodermal-derived cancers, but occurring late in endodermal-derived cancer [5]. The picture has also become increasingly complicated. The classical view was that *TP53* was a tumour-suppressor gene, and therefore, mutations leading to loss of function would be carcinogenic. More recently, it has emerged that *TP53* can also protect cancer cells, and therefore, mutations leading to gain of function could also lead to malignant transformation [5,7]. In the absence of cellular stress, the TP53 protein is short-lived and not detected by immunohistochemistry, but the product of mutated *TP53* may be degraded more slowly, and positive staining for the TP53 protein was therefore often assumed to suggest mutation [8]. At the time of our study, around the turn of the millennium, published reports indicated that *TP53*-mutations were frequent in OSCC (around 35%) and it had been noted that expression of TP53 was not an indicator of *TP53*-mutation in these cancers [9,10,11]. There were some studies finding common expression of the TP53 protein in hyperplastic and dysplastic oral lesions, but samples from lichen planus were rarely included, and mutation analyses were very limited.

We studied tissue samples from 150 oral mucosal lesions; one-third from OLP, one-third from oral leukoplakia and one-third from OSCC. We performed immunohistochemical staining for the TP53 protein and *TP53*-mutation analysis. The aim was to shed some light on the role of TP53 in oral carcinogenesis [12] The original study was followed up seven years later [13], and in addition, a separate study was performed on eight patients with OSCC and pre-malignant oral lesions for whom a series of samples over several years was available [14]. These three studies will be briefly summarized below and results added from a current follow-up. The study will be discussed in light of recent literature.

## 2. Summary of Three Studies on OLP and OSCC in Iceland

### 2.1. The Original Study of TP53 in OSCC and Premalignant Oral Lesions

For the original study group, 150 samples from oral squamous cell carcinoma (OSCC) and potentially premalignant oral lesions were obtained from the Department of Pathology, University of Iceland, which covers the whole of the Icelandic population, around 260,000 at the time of the study. Included were all cases of OSCC and hyperkeratosis (clinically leukoplakia) in the years 1990–1995 (55 and 47 samples, respectively) and all 48 samples from oral lichen planus (OLP) from the years 1985–1995. All samples were reviewed by the pathologist who co-authored the paper (Johannes Björnsson) to confirm the diagnosis. Staining for the TP53 protein was performed using standard immunohistochemical methods and graded on a scale of 1 to 6, where grades 1 and 2 (strong to moderate staining in >50% of nuclei) were classified as positive, and weak or absent nuclear staining and cytoplasmic staining was defined as negative. Mutation analysis was done by constant denaturation gel electrophoresis (CDGE), screening for mutations in hotspots A, B, C, and D, and Exon 6.

**Figure 1:**
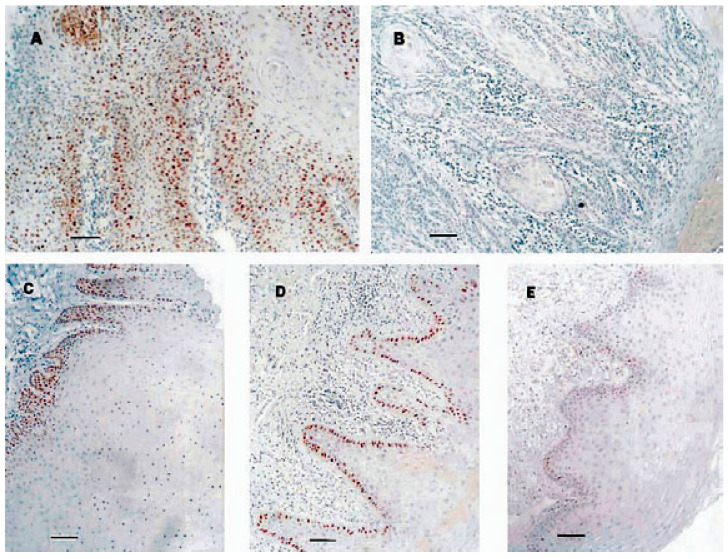
Expression of the TP53 protein in oral squamous cell carcinoma (OSCC), hyperkeratosis, lichen planus, and normal mucosa. (**A**) OSCC, positive staining, grade 1 (see text). (**B**) OSCC, negative staining. (**C**) Hyperkeratosis, positive staining, grade 2; note spreading of positive nuclei into suprabasal area. (**D**) Lichen planus, positive staining, grade 2; note that staining is confined to basal layer. (**E**) Normal mucosa, scattered staining, grade 4; scattered positive nuclei in basal layer. Bar, 50 mm. Reproduced with permission.

In this study, the results for already-diagnosed cases of OSCC were as expected. Strong positive staining for the TP53 protein was seen in over half of these (Figure 1) and one out of five was mutated, but mutations were not necessarily associated with positive protein staining. Mutations were identified in Exon 8 Hsp D in four cases and Exon 7 Hsp D in two cases, with one of these having mutations in both; Exon 6 in another four cases and one case each with mutations in Exon 5 Hsp A and Exon 5 Hsp B. The samples with hyperkeratosis (HK) had a low rate of positive TP53-protein staining (13%) and mutations (three out of twenty tested, in Exon 5 Hsp A (two cases) and Exon 8 Hsp D). Over half of these samples showed no nuclear expression of the TP53 protein. The unexpected results occurred in the group diagnosed with OLP where only two samples showed complete absence of the TP53 protein and one-third were classified positive for expression of the TP53 protein. This was confined to the basal layer (Figure 1), as had been noted in previous publications [15]. Microdissected positive nuclei from positive samples and an equal number of weakly staining samples (grade 3, classified as negative) were screened for *TP53* mutations, revealing a mutation rate of 33% (nine out of twenty-seven tested). Only a quarter of strongly positive cases were mutated, compared with one-third of weakly staining samples. The mutations occurred in Exon 8 Hsp D (three cases), Exon 5 Hsp A (three cases), Exon 6 (two cases), and Exon 7 Hsp C (one case). The strong protein expression can therefore be interpreted mainly as indicating response to cellular stress. In marked contrast, in samples with HK, usually regarded to have greater malignant potential than OLP, positively staining nuclei were seen in the suprabasal layer in the few positive samples. Of the three mutations identified in HK, one occurred in a TP53-protein positive sample.

The conclusion from this study was that the unexpectedly high rate of *TP53* mutations in OLP was remarkable and that the mutations might identify a subgroup of patients with a higher risk of developing OSCC that should be kept under close follow-up.

### 2.2. Longitudinal Study of Eight Patients with OSCC and Premalignant Oral Lesions

Finding this high rate of *TP53* mutations in OLP called for further study of the risk of malignant transformation and the time scale for such development. Thus, a study was performed on eight selected patients who had been under long-term review (two to twelve years) with one of the authors (WPH) because of oral lesions clinically regarded as suspicious of malignant potential, and from whom multiple biopsies [2,3,4,5] were available. Four of these patients were included in the original study group. Only one patient had OSCC when the first sample was taken from a lesion clinically diagnosed as erythroplakia. This sample was not mutated. A second sample taken two years later was mutated, but the lesion was not malignant. Another sample taken a few months after that from a different site had the same mutation and was diagnosed as OSCC. Five of these eight patients had lesions that were clinically diagnosed as resembling lichen planus, but histology confirmed this diagnosis in only two cases. Both of these were *TP53*-mutated and included in the original study. One of these two patients was diagnosed with OSCC three years later, the other has remained cancer-free. Four of the seven originally cancer-free patients developed OSCC and they were all *TP53*-mutated. From this study, it was concluded that *TP53* mutation can occur early or late in OSCC development.

### 2.3. Follow-Up Study and Current Follow-Up of Original Group

A follow-up study was performed on the original study group ending in March 2008, giving a follow-up time of 11–17 years (mean 13.3). Sufficient information was available for 144 of the original group of 150: 54 cases of OSCC and 45 each of HK and OLP. Follow-up was performed by searching for and reviewing all subsequent reports from the Department of Pathology, and by obtaining information on survival and cause of death from Statistics Iceland. Disease progression and recurrence in OSCC patients was not related to *TP53* mutations. Among the patients with hyperkeratosis, the lesion had progressed to OSCC in one patient who was among the seventeen confirmed mutation-free cases. The OLP patient already identified with OSCC in the longitudinal study was one of the nine *TP53-*mutated cases. In this patient, the cancer occurred in a tonsil, not the site of the original OLP lesion. The original sample from this patient had three *TP-53* mutations, in hotspots C and D, and exon 6. Two subsequent samples from local recurrences had a mutation in hotspot C. For the current publication, a search was performed for further entries for these 144 patients in the Icelandic Cancer Registry. This revealed one further case of OSCC in the lip in a patient with OLP 21 years after the first sample was taken, again in a different site from the original lesion. In this case, the *TP53*-mutation was in hotspot A. Both these cases were *TP53*-mutated, but only the second was positive for the TP53 protein. A summary of follow-up of the original OLP cases is shown in Table 1.

## 3. Discussion

Our original study was based on 45 OLP patients diagnosed between 1985–1995. With the latest follow-up in 2021, our study shows that out of nine patients who had *TP53* mutations in their original OLP lesion, two developed OSCC after delays of 6 and 21 years, respectively, albeit in different sites. This result lends support to the view that OLP is a premalignant lesion and that the malignant potential is linked to mutation and loss of function of *TP53*. It also emphasizes that the time lag can be very long and brings to mind the concept of field cancerization.

### 3.1. The Malignant Potential of OLP

The abstract of a recent paper on a Finnish study of a cohort of 13,100 women starts with a very familiar sentence, noting that “the association of lichen planus (LP) and cancer has been under debate for decades”. Remarkably, LP was associated with an increased risk of cancer of the lip, tongue, and oral cavity, as well as of the oesophagus, larynx, and vulva, but this was not true for cancer of the pharynx, vagina, and skin. The standardized incidence ratio was highest for lip, oral cavity, and tongue: five, eight, and twelve, respectively. The risk remained significant with follow-up ≥5 years [3]. The authors conclude that a diagnosis of LP is significantly associated with cancer in the sites mentioned, and, importantly, that the multisystem character of the disease calls for a multidisciplinary approach. The systematic review study of Fitzpatrick et al. published in 2014 found that among 7806 OLP patients in 16 studies, there was a significantly increased risk of malignant transformation with a slight female predominance, with the tongue being the most common site [16]. It was concluded that OLP patients should be observed regularly even if they do not fit traditional high-risk categories for oral cancer. The more recent systematic review by Giuliani et al. from 2018 based on 21 reports on 6559 patients concluded that the increased risk of malignant transformation of OLP was associated with the erosive type, female gender, and tongue site [2]. The authors called for strict clinical and histological diagnostic criteria for OLP, thus emphasising an important reason for the long-standing discussion on this matter. Aghbari et al. performed a meta-analysis of the data of 20,095 patients from 57 studies and found a slight but significant pooled risk of 1.1% that was associated with other known risk factors [1]. Richards from the Centre of Evidence-Based Dentistry in Dundee, Scotland, summarized these three review studies [17] and found that they provided some “evidence to confirm the potential malignant status of OLP”. He also pointed out some of their potential weaknesses; they were limited to English and the studies included might have suffered from selection and recall bias, retrospective approach, and missing data. Richards concluded, finally, that “in order to properly investigate the potential for malignant transformation of OLP long term multicentre cohort studies with good clinical and histological information are needed”. Few prospective cohort studies of OLP patients have been reported. Bombecarri et al. followed a cohort of 327 OLP patients in Northern Italy for a mean of 81.7 months (up to 108 months) and detected 8 cases of OSCC occurring after 23 to 62 months, with an annual rate of malignant transformation of 0.36% [18]. This can be compared with the transformation rate of monoclonal gammopathy of undetermined significance (MGUS) with annual progression to multiple myeloma of 1% [19]. Both cases in our study occurred after much longer time than observed by Bombecarri et al.

### 3.2. Developing Knowledge on TP53

TP53, or p53, as it was known for a long time, was discovered as a cancer-associated protein in 1979. It was originally erroneously classified as an oncogene, but when it was cloned in 1983, it was realized to be a tumour-suppressor gene [20], and this was then attributed to its function as “guardian of the genome” [6]. It is the most commonly mutated gene in a wide variety of cancers [5]. At the time of our study, the main the emphasis was on the role of TP53 in protecting the genome i.e., responding to DNA damage or stress linked directly to DNA damage, thus eliminating cells carrying carcinogenic mutations. In the decades after its discovery, extensive research has shown that TP53 belongs to a small family of DNA-binding proteins that are important in invertebrates for maintaining a healthy germ line and preventing mutations in offspring [5]. In long-lived vertebrates, these proteins have been adapted to serve the same purpose in tissue-specific stem cells. *TP53* interacts with the *MDM2* gene, and the *TP53/MDM2* hub is now realized to be central to the response to a great variety of cellular stresses, including DNA damage, telomere erosion, metabolic stress, hypoxia, oxidative stress, and more [5]. It has also become clear that some stress signals, such as infections and inflammation, can lead to inhibition of TP53. The final cellular response varies according to the type of stress [5]. The majority of *TP53* mutations occurring in cancer lead to loss of the protective function of the wild-type protein, but it is now realized that missense mutations can result in altered protein interactions or gain-of-function mutations, leading to aberrant stress responses [7]. The tissue context is important. Cancer-causing mutations are selected for fitness in stem cells in ecto- and mesodermal- derived tissues, whilst in endodermal-derived tissues these mutations may be dormant and selected for at a later stage in tissue-specific stem or progenitor cells [5]. Furthermore, in endodermal-derived cancer, e.g., colon cancer, *TP53*-mutation is often the last decisive event following a series of other mutations [21]. In our two OLP patients who developed OSCC, the *TP53* mutation occurred very early. When the original lesion was diagnosed as hyperplasia, *TP53* mutation was an early event in one of the four cases.

### 3.3. Searching for Risk Factors That Contribute to Malignant Transformation of OLP

Since malignant transformation of OLP is a rare event, there is a great need to define factors that enhance this risk in order to define a specific subgroup that would benefit from targeted clinical observation. Two main approaches are used in the search for markers for malignant progression: targeted testing for expression of candidate proteins and non-targeted screening for genetic changes. Altered expression of proteins involved in cell proliferation (Ki-67), cell cycle control (p21), apoptosis (Bcl-2 family), and inflammation (Cox2, MMP’s) has been reported in OLP, and increased expression of TP53 has been noted very frequently [4,22]. Many of these proteins, especially TP53, show increased expression in a large proportion of OLP lesions, and testing for them is therefore more likely to support a diagnosis of OLP, rather than to identify a subgroup that is at risk to progress to cancer. Agha–Hosseini et al. concluded in their literature review of non-genetic and genetic risk factors that malignant transformation was most frequently observed in erythematous and erosive lesions, but state no conclusion for reported protein markers [4]. Recurrent DNA copy number changes have been reported in OSCC but data on OLP are very limited. In a recent study, Németh et al. found no such changes in OLP, but, interestingly, they identified several changes in DNA methylation patterns in OLP that were also seen in OSCC [23]. Taken together, no measurable markers have been identified to be useful in detecting OLP patients who may be at risk for progression to OSCC. Expression of the TP53 protein is common in OLP and most likely reflects cellular stress in a chronic inflammatory condition. We are not aware of other studies apart from our own that screen for *TP53* mutations.

### 3.4. Field Cancerization

The concept of field cancerization was first put forward by Slaughter in 1953 and was based on his observations on oral cancer developing and recurring in different sites in the oral cavity [24]. The phenomenon was subsequently observed in several other tissues, including lung, esophagus, colon, vuvla, cervix, breast, and bladder, and underlying acquired genetic changes in stem cells were identified [25]. This gave rise to the definition formulated by Braakhuis et al.: “the presence of one or more areas consisting of epithelial cells that have genetic alterations. A field lesion (or shortly ‘field’) has a monoclonal origin, and does not show invasive growth and metastatic behavior, the hallmark criteria of cancer” [25]. Genes identified as drivers for field cancerization include members of the Notch family, but *TP53* mutations play a major role [26]. The initial effect may be very subtle and survival advantage of *TP53*-mutated cells may be evident only under conditions of stress, as noted in skin exposed to UV light [27]. In lichen planus, the stress factor would be chronic inflammation, as has been observed in the colon, where expansion of a *TP53*-mutant clone occurs only in the presence of inflammation [26,28]. Interactions of the mutant clone with the microenvironment are important for its growth advantage leading to expansion of the cancerized field [26]. Panels of molecular markers have been developed to aid prediction for malignant progression in known cancer-predisposing conditions such as Barrett’s oesophagus [29]. Field cancerization is a long evolutionary process, and therefore, the identification of an aberrant clone may have limited predictive power as such, but detection of clonal expansion has been found to have prognostic value [26]. The long delay and widespread location observed in our two OLP-derived OSCC are in line with a *TP53*-mutation occurring in a stem cell leading to a cancerized field in the presence of inflammation.

## 4. Conclusions

Before concluding, a note should be added on the long-standing discussion on diagnostic criteria for OLP. The currently accepted WHO criteria are based on the paper published by van der Meij and van der Waal in 2003 [30], i.e., after recruitment for our studies. As noted above, all diagnoses in our study were based on histology and reviewed specifically by one pathologist, using accepted criteria. Obviously it is not possible to draw conclusions from such a small study, but if we imagine that the figures were scaled up, there would have been two conclusions from our original publications that, at the time, were somewhat paradoxical or contrary to what was commonly believed. Firstly, oral lichen planus did not appear to be a particularly strong risk factor for oral cancer, since OSCC had developed in one out of forty-five, the same rate as observed for patients with hyperkeratosis. Secondly, *TP53* mutations did not indicate a high risk of developing cancer, with one case of OSCC out of nine with mutation, but follow-up samples had also shown that the *TP53* mutation persisted without cancer development in another case. One additional case of OSCC diagnosed 7 years after the end of the study forces us to reconsider our conclusion, and the balance is now tipped towards supporting a risk of OSCC developing in OLP patients, and towards this risk being associated with *TP53* mutation. Recent literature suggests that a large number of retrospective studies have not provided a definitive conclusion and consensus, and that there is a need for large multicentre prospective studies. Testing for expression of the TP53 protein is not likely to indicate if OLP patients are at increased risk of developing cancer, as this is very common and indicative of cellular stress rather than presence of mutations. Screening for *TP53* mutations could help to identify the subgroup of OLP patients that is most likely to progress to cancer, and would therefore require close clinical observation.

## Figures and Tables

**Table 1:** Follow-up of patients with Oral lichen planus related to TP53 protein expression and *TP53* mutations.

TP53 Protein Expression	*TP53* Mutation	OSCC/Site/Years Until Diagnosis
Positive: 16	Yes: 4	1/lip/21
	No: 12	
	Not done: 0	
Negative: 29	Yes: 5	1/tonsil/6
	No: 9	
	Not done: 15	

## Data Availability

Not relevant.
